# Current progress in dengue vaccines

**DOI:** 10.1186/1423-0127-20-37

**Published:** 2013-06-13

**Authors:** Shu-Wen Wan, Chiou-Feng Lin, Shuying Wang, Yu-Hung Chen, Trai-Ming Yeh, Hsiao-Sheng Liu, Robert Anderson, Yee-Shin Lin

**Affiliations:** 1 Department of Microbiology and Immunology, National Cheng Kung University Medical College, Tainan, Taiwan; 2 Center of Infectious Disease and Signaling Research, National Cheng Kung University, Tainan, Taiwan; 3 Institute of Clinical Medicine, National Cheng Kung University Medical College, Tainan, Taiwan; 4 Department of Biochemistry and Molecular Biology, National Cheng Kung University Medical College, Tainan, Taiwan; 5 Department of Medical Laboratory Science and Biotechnology, National Cheng Kung University Medical College, Tainan, Taiwan; 6 Departments of Microbiology & Immunology and Pediatrics, and Canadian Center for Vaccinology, Dalhousie University, Halifax, Nova Scotia, Canada

**Keywords:** Dengue, Immunopathogenesis, Vaccine

## Abstract

Dengue is one of the most important emerging vector-borne viral diseases. There are four serotypes of dengue viruses (DENV), each of which is capable of causing self-limited dengue fever (DF) or even life-threatening dengue hemorrhagic fever (DHF) and dengue shock syndrome (DSS). The major clinical manifestations of severe DENV disease are vascular leakage, thrombocytopenia, and hemorrhage, yet the detailed mechanisms are not fully resolved. Besides the direct effects of the virus, immunopathological aspects are also involved in the development of dengue symptoms. Although no licensed dengue vaccine is yet available, several vaccine candidates are under development, including live attenuated virus vaccines, live chimeric virus vaccines, inactivated virus vaccines, and live recombinant, DNA and subunit vaccines. The live attenuated virus vaccines and live chimeric virus vaccines are undergoing clinical evaluation. The other vaccine candidates have been evaluated in preclinical animal models or are being prepared for clinical trials. For the safety and efficacy of dengue vaccines, the immunopathogenic complications such as antibody-mediated enhancement and autoimmunity of dengue disease need to be considered.

## Review

### Introduction

Dengue virus (DENV) is a member of the *Flavivirus* genus of the *Flaviviriade* family which also includes yellow fever virus (YFV), West Nile virus (WNV), Japanese encephalitis virus (JEV) and tick-borne encephalitis virus. There are four antigenically distinct serotypes (DENV1-4) based on neutralization assay. DENV is transmitted to humans mainly by Aedes mosquitoes (*Aedes aegypti* and *Aedes albopictus*) [1]. The prevalence of dengue disease is high especially in the Asia-Pacific region and the Americas. All four DENV serotypes are now circulating in these areas. With increased international travel and climate change, people are at risk of dengue infection beyond the traditional tropical and subtropical areas. Dengue disease is becoming one of the most important emerging vector-borne viral diseases. An estimated 50 million dengue infection cases occur globally with around 500,000 cases of severe dengue and 20,000 deaths per year [2].

### Characteristics of dengue virus

DENV is a lipid-enveloped positive-strand RNA virus. The RNA genome of DENV is about 10.7 kb and encodes three structural proteins, namely capsid protein (C), precursor membrane/membrane protein (PrM/M), and envelope protein (E). Besides the structural proteins, there are seven nonstructural proteins (NS) which are associated with viral replication and disease pathogenesis. The coding of the viral proteins is organized in the genome as C-prM-E-NS1-NS2A-NS2B-NS3-NS4A-NS4B-NS5 [3,4].

The C protein stabilizes the viral RNA within the viral nucleocapsid. The N-terminus of the C protein encodes a nuclear localization sequence which allows C protein translocation into the nucleus and interaction with heterogeneous nuclear ribonucleoprotein [5]. A recent report showed that DENV C protein may interact with human death domain-associated protein Daxx and induce apoptosis [6]. The prM protein acts as a chaperone that helps the folding of E protein. The M protein is a proteolytic fragment derived from its precursor form prM by furin cleavage in the trans-Golgi network. The E protein is the major protein exposed on the virus surface and has three distinct structural domains. Domain I of the E protein is an eight-stranded β barrel and is structurally positioned between domain II and domain III. Domain II contains a dimerization region and a highly conserved fusion loop. Domain III consists of an immunoglobulin-like fold and is the proposed receptor binding domain [4,7].

The NS1 protein is a glycoprotein and is expressed in three forms: an endoplasmic reticulum (ER)-resident form, a membrane-anchored form, and a secreted form. In DENV-infected mammalian cells, NS1 is synthesized as a soluble monomer and is dimerized after posttranslational modification in the ER where it plays an essential role in viral replication (ER-resident form) [8,9]. In infected mammalian cells, secreted NS1 (sNS1) exists as hexamers, and is another dominant target of humoral immunity [10]. Soluble NS1 binds to the plasma membrane of uninfected cells by interactions with heparin sulfate and chondroitin sulfate E [11]. NS1 is expressed on the surface of infected cells as membrane-anchored form, possibly by a glycosylphosphatidyl inositol (GPI) anchor [12] or lipid raft association [13]. The functions of surface expressed NS1 have been reported to include signal transduction [12] and complement activation [11]. In addition, N-linked glycosylation of NS1 modulates its secretion, cell-surface expression, hexamer stability, and interaction with human complement [14].

NS2A can cleave itself from NS1 by its protease activity and at the same time properly processes NS1 in the ER [15]. NS2A is also capable of blocking interferon (IFN)-mediated signal transduction [16]. NS2B is a cofactor of NS3 which together form an active serine protease complex [17]. NS3 is a multifunctional protein with N-terminal protease domain, RNA 5′-triphosphatase, RNA helicase and RNA-stimulated NTPase domain in the C-terminal region. Protease activity is required to process the polyprotein precursor and is essential for viral replication [18]. The helicase activity of NS3 is involved in viral replication and viral assembly [19,20]. Both NS4A and NS4B may be involved in blocking IFN-α/β-induced signal transduction [16,21]. However, NS4B can modulate viral replication by its interaction with NS3 [22]. NS5 is the largest and most conserved DENV protein. It encodes two distinct enzyme activities, i.e. *S*-adenosyl methyltransferase which can methylate the 5′end of viral RNA [23] and RNA-dependent RNA polymerase [24].

### Dengue disease manifestation and classification

Infection with DENV may cause mild DF with an onset of fever accompanied by severe headache, retro-orbital pain, myalgia, arthralgia, abdominal pain, rash, and minor hemorrhage in the form of petechiae, epistaxis, or gingival bleeding. Leukopenia and thrombocytopenia may occasionally be observed in DF patients [25]. Severe DHF/DSS generally occurs in those patients who are secondarily infected with a different DENV serotype. However, DHF/DSS may still occur in primary infection [26]. DHF is characterized by all the symptoms of DF but also shows severe bleeding, thrombocytopenia, plasma leakage and organ involvement. Traditionally, there are four grades for classifying DHF. Grades I and II of DHF represent relatively mild cases without shock, whereas grades III and IV of DHF are more severe and might develop to disseminated intravascular coagulation [27,28]. The WHO recently reclassified dengue due to difficulties in applying the old classification in the clinical situation [29,30]. The new guidelines focus on levels of disease severity and cases are classified as dengue without warning sign, dengue with warning sign, and severe dengue. Patients with severe plasma leakage, severe bleeding or severe organ involvement are defined as severe dengue. The new classification system emphasizes the early recognition of potential manifestations. Therefore, recent reports indicate higher sensitivity using the revised classification [31]. Early prediction is very important to avoid unnecessary hospitalization, especially in hyper-endemic areas. However, whether the new guidelines reduce the specificity of dengue confirmation needs to be further evaluated.

### Dengue virus pathogenesis

The complicated pathogenesis of DHF/DSS is not fully resolved. However, several hypotheses for the pathogenesis of DENV infection have been proposed, including viral pathogenesis and immunopathogenesis, which play significant roles in major manifestations of DHF/DSS, such as hemorrhage, thrombocytopenia, plasma leakage and organ impairment [32-35].

#### Viral pathogenesis

Viral pathogenesis indicates the pathology directly caused by the virus which is subject to serotypic or genotypic differences. Differences in transmission efficiency and disease expression between the four serotypes are still uncertain, but DENV-2 and DENV-3 have been associated with an outbreak of severe dengue [36]. Viral genetic and structural differences might contribute to virus variation and influence human disease severity [37-40]. The affinity of DENV for host receptors might affect virus infectivity as well as virulence. Recent studies indicated that certain mutations in the E and NS3 proteins altered the virulence of DENV by enhancing binding activity to host cells and increasing viral replication [41].

#### Immunopathogenesis

The critical phase of dengue disease is not observed at the peak of viremia but rather when the viral burden has started to decline. This has led to the suggestion that immune responses are not only responsible for virus clearance but also contribute to pathogenesis. The adaptive immune responses, inflammatory mediators and autoimmunity are important factors involved in immunopathogenesis [34,42].

The adaptive immune responses play important roles in the immunopathogenesis of dengue disease. Antibody-dependent enhancement (ADE) is a well-known phenomenon of dengue pathogenesis. From epidemiological studies, the presence of preexisting heterologous antibodies (Abs) which fail to neutralize the current infecting serotype is a major factor for developing DHF/DSS in both infants and adults [43]. Those sub-neutralizing Abs enhance viral uptake by Fcγ receptor (FcγR)-dependent [43] or FcγR-independent mechanisms [44]. Recently, a new hypothesis (termed intrinsic ADE) postulates that FcγR-mediated DENV internalization suppresses innate immunity, increases interleukin-10 production and biases T-helper-1 (Th1) responses to Th2 responses, leading to both high levels of viral load and Abs in dengue patients [45-47]. In addition to neutralizing and infection-enhancing Abs, memory T cells which cross-react with heterologous viruses could provide partial protective immunity, as well as cause immunopathology [48]. Secondary dengue infections show predominant expansion of T cells with low affinity for the current infecting serotype and high affinity for the previously infected DENV serotype (known as original antigenic sin) [49]. Numerous studies showed that the cross-reactive T cells produce high concentrations of inflammatory cytokines which might correlate with plasma leakage in severe dengue [50-53]. DENV-specific human CD4^+^ cytotoxic T cell clones have been demonstrated to not only produce cytokines but also lyse bystander target cells *in vitro*[54].

Several studies have shown that patients with severe dengue have elevated plasma markers, such as cytokines, chemokines, soluble receptors, coagulation and endothelial markers [27,55-57]. The abnormal production of plasma markers mainly comes from monocytes [58], T cells [50-53], mast cells [59], and neutrophils [60]. High levels of C3a and C5a have also been measured in dengue patients’ plasma [11,61]. In addition, complement could be activated by soluble NS1 and anti-DENV NS1 Abs on DENV-infected endothelial cells [61,62].

Autoantibodies and molecular mimicry represent another contributory factor in dengue disease pathogenesis. Autoantibodies against platelets [63-65], endothelial cells [66,67] and coagulatory molecules [66,68-70] have been observed in dengue patient sera and associated with severe dengue. Molecular mimicry between platelets, endothelial cells or coagulatory molecules with NS1, prM and E proteins may explain the cross-reactivity of anti-NS1, anti-prM or anti-E Abs to host proteins. The consequences arising from these cross-reactive Abs include platelet dysfunction, endothelial cell apoptosis, coagulation defect, and macrophage activation [42,57,71,72]

### Protective immune responses

Both humoral and cellular immunity contribute to DENV clearance and protection. The E protein is the major component on the surface of DENV virion and is a dominant target of Ab responses against DENV. E protein binds to cellular receptors and mediates fusion between viral envelope and cellular membrane during viral entry [55,73-75]. Passive immunization with anti-E Abs provides protection against DENV infection in mice [76]. Although the NS1 protein is not a component of the virion, the NS1 protein is expressed on the surface of infected cells [12] and is secreted into the circulation [10]. Abs against NS1 can trigger complement-mediated lysis of DENV-infected cells *in vitro* and protect mice from DENV challenge [77]. In addition, monoclonal Abs against prM/M have been shown to provide protection against DENV challenge [78].

Infection with DENV results in the development of CD4^+^ and CD8^+^ T cell responses against multiple viral proteins, of which the NS3 protein appears to be immunodominant [79]. The effector functions of DENV-specific T cells include cytokine production and target cell lysis [55]. Both DENV-specific CD4^+^ and CD8^+^ T cells protect mice from DENV infection; however CD8^+^ T cells are more efficient [80-83]. Recent studies further demonstrated that both cross-reactive B and T cells provide protection against a secondary heterotypic DENV infection [84,85].

### The challenges of dengue vaccine development

The ideal dengue vaccine should provide long-term homotypic and heterotypic protection. Therefore, there are several factors which require consideration. First, the vaccine must be protective against each of the four DENV serotypes to reduce the risk of ADE. Second, the immunization should be safe and not cause unacceptable side-effects caused by cross-reactive Abs or cross-reactive T cells. Third, the cost should be affordable to the individuals who most need the vaccines [86,87]. There are still several obstacles for the development of dengue vaccines. One is that the complicated pathogenesis is not fully understood. Another hindrance is the lack of suitable animal models. DENV can infect nonhuman primates but does not replicate well or cause marked disease. For reasons of cost and convenience, mouse models have been used to test vaccine candidates prior to testing in nonhuman primates. In general, immunocompetent mice are the more suitable models to test the immunogenicity of a vaccine. However, DENV replicates poorly in these mice. Recent progress has been made in modeling dengue in mice, using transgenic, knockout and humanized approaches [88]. One recently described mouse model explored the use of intravenous, intraperitoneal, intracerebal or intradermal inoculation of DENV, resulting in liver pathology, neurological symptoms, thrombocytopenia, or hemorrhage [89,90]. In addition, the SCID-tumor mouse model has been tested for live-attenuated dengue vaccine [91] and the immunocompromised mouse model AG129 has been developed for vaccine testing [92].

### Current vaccine progress

Although no licensed dengue vaccine is yet available, several vaccine candidates are under development. These include live attenuated virus vaccines, live chimeric virus vaccines, inactivated virus vaccines, and live recombinant, DNA and subunit vaccines [93]. Live viral vaccines have advanced to clinical trials, but have shown problems, such as unequal immunogenicity of the four serotypes and viral interference among the four serotypes in tetravalent formulations. Non-viral vaccines have also been proposed and developed for safety reasons. This includes subunit vaccines that mostly focused on the E protein or its derivatives. However, the difficulty of eliciting balanced levels of neutralizing Abs to each of the four serotypes remains a major concern. NS1 is another subunit vaccine candidate that it is not a virion-associated protein and it has no ADE effects [30].

#### Live attenuated virus vaccines

Live attenuated virus vaccines contain weakened viruses that still can induce adaptive immune responses to both structural and nonstructural proteins. The replication of live attenuated viruses should be sufficiently restricted to avoid pathological effects. One of the most successful examples of a live attenuated virus vaccine is the 17D strain of YFV, another member of the flavivirus family [94]. Unfortunately the search for an equally successful attenuated dengue vaccine has proven more elusive. In pre-clinical study, live attenuated viruses derived from serially passaged DENV in primary dog kidney (PDK) cells were inoculated in rhesus monkeys to test for viremia and immune responses [95]. Investigators at Mahidol University in Bangkok, Thailand and the Walter Reed Army Research Institute (WRAIR) group in the USA independently developed attenuated DENV vaccine candidates by passage in tissue culture cells for each serotype of DENV [96,97]. Tetravalent dengue vaccine formulations produced by the Mahidol group were used for Phase I and II clinical trials in Thai adults and children. Not all of the volunteers seroconverted to all four DENV serotypes and some showed unacceptable reactogenicity. Consequently, further clinical testing was stopped [98-100]. The WRAIR-produced tetravalent dengue vaccine formulation also showed problems of unbalanced immunogenicity and reactogenicity [97]. New formulations seem to be safe and immunogenic in a Phase II study, however, the protective efficacy needs to be further evaluated [101].

A more modern approach is based on site-directed mutagenesis of the viral genome to cause attenuation. A deletion of 30 nucleotides (∆30) in the 3′-untranslated region of DENV4 was first demonstrated to attenuate DENV4, named as DEN4∆30 [102], and used in Phase I clinical evaluation [103]. However, while this strategy resulted in attenuation for DENV1 and DENV4, with retained immunogenicity, it was less successful for DENV2 and DENV3 [102,104-106]. Hence, an alternative chimeric strategy for DENV2 and DENV3 was designed using the ∆30DEN4 as genetic backbone for DENV2 and DENV3 (designated as DEN2/4∆30 and DEN3/4∆30). These monovalent DENV vaccines (DEN1∆30, DEN2/4∆30, DEN3/4∆30 and DEN4∆30) have been tested for attenuation and immunogenicity in animal models and humans, and the attenuated tetravalent DENV vaccine admixtures are currently in Phase I clinical studies [30,107].

#### Live chimeric virus vaccines

The most advanced product so far, Sanofi Pasteur’s ChimeriVax Dengue tetravalent vaccine (CVD1-4) utilized the licensed YFV 17D vaccine as backbone, each expressing the prM and E genes of one of the four DENV serotypes. Pre-clinical studies demonstrated that the tetravalent vaccine is genetically and phenotypically stable [108,109], less neurovirulent than YFV 17D [110], and immunogenic in monkeys [111]. In Phase I studies, the tetravalent CVD vaccine appeared safe with relatively low viremia [112-114]. Recently, however, Phase II study showed only 30 percent effectiveness and efficacies against only DENV1, 3 and 4 serotypes [115]. These results indicate that the Sanofi dengue vaccine still carries the risk of ADE and needs more testing, modification and/or clinical trials especially in dengue-endemic countries [116].

#### Inactivated virus vaccines

Inactivated virus vaccines have two advantages over live virus vaccines, i.e. no possibility of reverting to virulence (safety) and relative ease of inducing balanced immune responses (for tetravalent vaccines). However, some challenges remain, such as lack of the immunity to NS proteins and a requirement of adjuvants for enhancing immunogenicity. A purified, inactivated DENV2 vaccine has been shown to be immunogenic and protective in mice and rhesus monkeys [117] as well as formulated with adjuvants for inducing higher levels of neutralizing Abs and protection against viraemia [118].

#### Live recombinant, DNA and subunit vaccines

Recent advances in molecular biology have spurred dengue vaccine efforts using live recombinant, DNA and subunit vaccines. Generally, the DENV E protein is used as the major immunogen. Certain live viral vectors, such as adenovirus, alphavirus and vaccinia virus are designed for direct administration to the host and have been engineered to express DENV E protein for further evaluation as vaccines [119-121]. In addition, recombinant E proteins expressed from yeast and insect cells have been used to test for immunogenicity [122-124] and protective efficacy [124,125] in animal models. Truncated E proteins (DEN-8E) produced for all serotypes have been developed with aluminum hydroxide (adjuvant) as tetravalent vaccine formulations [125].

The domain III of the DENV E protein (EDIII) is the proposed receptor binding domain and elicits neutralizing Abs [30]. It has been demonstrated that immunization with recombinant EDIII induces protective Abs against DENV in both mouse [126] and nonhuman primate models [127]. To develop a tetravalent subunit vaccine, Leng et al. have prepared a consensus EDIII (cEDIII) protein by aligning amino acid sequences from different isolates of the four serotypes of DENV. They showed that the novel cEDIII successfully elicited cross-neutralizing Ab responses against four serotypes in a mouse model [128] and neutralizing Ab responses against DENV-2 in a nonhuman primate model [129]. An engineered heterologous lipoprotein (recombinant lipo-EDIII) which is EDIII protein fused with lipid signal peptides was reported to induce higher levels of neutralizing Abs than EDIII protein formulated with alum adjuvant [130]. Combining the tetravalent and adjuvant effects, a novel single-dose dengue subunit vaccine (lipo-cEDIII) was demonstrated to neutralize the four serotypes of DENV and induce memory immune responses [131]. However, recent studies indicated that EDIII-specific Abs do not constitute a large percentage in the dengue patient sera and only contribute to a small proportion of DENV neutralization *in vitro*[132-134]. Thus, the main epitope of DENV for neutralizing Abs in the human as well as the applicability of EDIII-based vaccines remain to be defined.

NS1 is not a structural component of the virion, and therefore, does not contribute to ADE. Anti-DENV NS1 Abs are potentially protective antibodies since they trigger complement-mediated lysis of DENV-infected cells [77]. Several studies indicated that passive immunization with anti-NS1 Abs [131], DNA vaccine against NS1 proteins [135-138], or recombinant vaccinia virus expressing NS1 [139] and active immunization with NS1 proteins [77,140] could provide protection in mice against DENV challenge (Table 1). However, anti-NS1 Abs still have some pathogenic effects as determined both *in vitro* and *in vivo* due to cross-reactivity with host proteins [57,71,141]. A safe dengue NS1-based vaccine would likely require the deletion or modification of cross-reactive epitopes. In our studies, we have tested the immunogenicity, pathogenic and protective effects of full-length DENV NS1 and NS1 lacking the C-terminal amino acid residues 271-352 (designated ∆C NS1). Results showed that the immunogenicity of ∆C NS1 was similar to that of full-length DENV NS1 but, most importantly, ∆C NS1 did not induce pathogenic effects as assayed by cross-reactivity or bleeding tendency [142]. In addition, passive immunization with anti-∆C NS1 Abs provides protection against DENV infection in a hemorrhagic mouse model (unpublished data). We are currently evaluating the application of ∆C NS1 protein for vaccine development.

**Table 1:** Summary of NS1 subunit vaccine applications in mouse models

**Approach**	**DENV inoculation**	**Mouse**	**Outcome**	**Reference**
Monoclonal anti-NS1 Abs	DENV2 (NGC) 100 IC_50_ i.c.	BALB/c	50-93% Survival	[135]
DNA vaccine:	DENV2 (PL046)	C3H/HeN	pD2NS1: 50% Survival	[136]
pD2NS1	10^7^ PFU i. p.		50% Morbidity	
pD2NS1 + pIL12			pD2NS1 + pIL12: 80% Survival	
			20% Morbidity	
DNA vaccine: pcTPANS1	DENV2 (NGC)	BALB/c	50% Survival	[137]
	4,32 log_10_PFU i.c.			
DNA vaccine: pcTPANS1*	DENV2 (NGC)	BALB/c	pcTPANS1: 96.7% Survival	[138]
pcENS1**	4,32 log_10_PFU i.c.		10%Morbidity	
			pcEN1: 86.7% Survival	
			27% Morbidity	
Vaccinia Virus:	DENV4 (H241)	BALB/c	67% Survival	[139]
vNS1-15% NS2a	100IC_50_ i.c.			
rNS1 + CFA (adjuvant)	DENV2 (NGC)	BALB/c	88% Survival	[77]
	Lethal dose i.c.		18% morbidity	
rNS1 + LT_G33D_ (adjuvant)	DENV2 (NGC)	BALB/c	50% Survival	[140]
	4,32 log_10_PFU i.c.		80% Morbidity	

*TPA human tissue plasminogen activator, a secretory signal sequence.

**pcENS1: encoding the C-terminal E protein plus the NS1 region.

*i.c* intracerebal, *i.p* intraperitoneal.

Another alternative approach is the production of fusion proteins, such as E-NS1 proteins expressed by *E coli*[143] and prM-E-NS1 proteins encoded by DNA vaccine [144] which provide protection in mice. However, the cross-protection against other serotypes needs to be further investigated.

## Conclusions

Although no licensed dengue vaccine is yet available, the ever-increasing knowledge of dengue pathogenesis, is providing more insights into improved vaccine design. Important aspects of dengue vaccine development include common features such as immunogenicity, reactogenicity and protective efficacy but also dengue-unique features such as the heterotypic nature of the virus, the risk of ADE and cross-reactivity with host proteins. Furthermore, all of these aspects should ideally be tempered with considerations of cost and stability.

## Competing interests

The authors declare that they have no competing interests.

## Authors’ contributions

All authors discussed and designed the concept. SWW, CFL, RA and YSL collected information and prepared the manuscript. SWW wrote the manuscript. All authors read and approved the final manuscript.
